# Deafness-associated tRNA^Phe^ mutation impaired mitochondrial and cellular integrity

**DOI:** 10.1016/j.jbc.2024.107235

**Published:** 2024-03-27

**Authors:** Xiaowan Chen, Feilong Meng, Chao Chen, Shujuan Li, Zhiqiang Chou, Baicheng Xu, Jun Q. Mo, Yufen Guo, Min-Xin Guan

**Affiliations:** 1 Department of Otolaryngology-Head and Neck Surgery, Lanzhou University First Hospital, Lanzhou, Gansu, China; 2 Institute of Genetics, Zhejiang University International School of Medicine, Hangzhou, Zhejiang, China; 3 Division of Medical Genetics and Genomics, The Children's Hospital, Zhejiang University School of Medicine, Hangzhou, Zhejiang, China; 4 Center for Mitochondrial Biomedicine, The Fourth Affiliated Hospital Zhejiang University School of Medicine, Hangzhou, Zhejiang, China; 5 Department of Otolaryngology-Head and Neck Surgery, Gansu Provincial Hospital, Lanzhou, Gansu, China; 6 Department of Otolaryngology-Head and Neck Surgery, Lanzhou University Second Hospital, Lanzhou, Gansu, China; 7 Department of Pathology, Rady Children’s Hospital, University of California School of Medicine, San Diego, California, USA; 8 Zhejiang Provincial Lab of Genetics and Genomics, Zhejiang University, Hangzhou, Zhejiang, China

**Keywords:** deafness, mitochondrial tRNA mutation, mitochondrial dynamics, mitophagy, apoptosis

## Abstract

Defects in mitochondrial RNA metabolism have been linked to sensorineural deafness that often occurs as a consequence of damaged or deficient inner ear hair cells. In this report, we investigated the molecular mechanism underlying a deafness-associated tRNA^Phe^ 593T > C mutation that changed a highly conserved uracil to cytosine at position 17 of the DHU-loop. The m.593T > C mutation altered tRNA^Phe^ structure and function, including increased melting temperature, resistance to S1 nuclease-mediated digestion, and conformational changes. The aberrant tRNA metabolism impaired mitochondrial translation, which was especially pronounced by decreases in levels of ND1, ND5, CYTB, CO1, and CO3 harboring higher numbers of phenylalanine. These alterations resulted in aberrant assembly, instability, and reduced activities of respiratory chain enzyme complexes I, III, IV, and intact supercomplexes overall. Furthermore, we found that the m.593T > C mutation caused markedly diminished membrane potential, and increased the production of reactive oxygen species in the mutant cell lines carrying the m.593T > C mutation. These mitochondrial dysfunctions led to the mitochondrial dynamic imbalance *via* increasing fission with abnormal mitochondrial morphology. Excessive fission impaired the process of autophagy including the initiation phase, formation, and maturation of the autophagosome. In particular, the m.593T > C mutation upregulated the PARKIN-dependent mitophagy pathway. These alterations promoted an intrinsic apoptotic process for the removal of damaged cells. Our findings provide critical insights into the pathophysiology of maternally inherited deafness arising from tRNA mutation-induced defects in mitochondrial and cellular integrity.

Mutations in mitochondrial DNA (mtDNA) have been associated with a wide spectrum of clinical presentations including neuromuscular disorders, diabetes, vision failure, and hearing loss (1, 2, 3, 4, 5). Human mitochondrial DNA (mtDNA) is a circular, double-strand molecule with 16.6 kb coding for two rRNAs and 22 tRNAs required for translation and 13 polypeptides for essential subunits of oxidative phosphorylation system (OXPHOS) (6, 7). In particular, the defects in mitochondrial RNA metabolism have been linked to sensorineural hearing loss that often occurs as a consequence of damaged or deficient inner ear hair cells (5, 8, 9, 10, 11). The m.1555A > G and m.1494C > T mutations in the 12S rRNA gene have been associated with both aminoglycoside-induced and nonsyndromic deafness (hearing loss is the only obvious medical problem) in many families worldwide (12, 13, 14, 15). The m.1555A > G and m.1494C > T mutations at the A site of ribosomes make the human mitochondrial ribosomes more bacteria-like and alter binding sites for aminoglycosides and mitochondrial translation (16, 17). The syndromic deafness (hearing loss with other medical problems such as diabetes)-associated tRNA mutations including the MELAS-associated tRNA^Leu(UUR)^ 3243A > G and MERRF-associated tRNA^Lys^ 8344A > G and maternally inherited diabetes and deafness (MIDD)-associated tRNA^Glu^ 14692A > G mutations occurred in heteroplasmic form (mutated mtDNA *versus* wild-type mtDNA) (18, 19, 20). The development of phenotypes arising from these mutations depends on the proportion of mutated mtDNA molecules in the cells, surpassing a threshold level to maintain full OXPHOS function in the vulnerability of tissue or organ (3). By contrast, the nonsyndromic deafness-associated tRNA mutations including the tRNA^Ser(UCN)^ 7445A > G, and 7511T > C, tRNA^His^ 12201T > C, tRNA^Asp^ 7551A > G, tRNA^Ile^ 4295A > G, tRNA^Cys^5783C > T and m.7516delA mutations were present in homoplasmy (21, 22, 23, 24, 25, 26, 27, 28). These mtDNA mutation(s) are by itself insufficient to produce the clinical phenotypes and other genetic and environmental factors such as nuclear modifier gene and aminoglycosides contributed to the development of clinical phenotype (2, 3, 8, 17, 18). These tRNA mutations have structural and functional consequences, including the processing of the tRNA from the primary transcripts, stability of the folded secondary structure, the charging of the tRNA, or the codon–anticodon interaction in the process of translation (20, 21, 22, 23, 24, 25, 26, 27). The aberrant tRNA metabolisms led to the impairment of mitochondrial protein synthesis, oxidative phosphorylation system (OXPHOS) and subsequently decline in ATP production required for the cochlear functions (29). However, the molecular mechanisms underlying these RNA mutations remain poorly understood.

Most recently, we identified a T to C transition at position 593 (m.593T > C) at the tRNA^Phe^ gene in a Chinese pedigree displaying late onset of nosyndromic deafness (30). As shown in Figure 1*A*, the m.593T > C variant changed U to C at position 17 of the DHU-loop, where the position is important for the structure and function of tRNA (31). Therefore, the m.593T > C mutation altered both the structure and the function of tRNA^Phe^. The aberrant tRNA^Phe^ metabolism may result in the impairment of mitochondrial translation, defective activities of OXPHOS complexes, diminished mitochondrial ATP levels, reduced membrane potential, and elevated production of reactive oxygen species. Subsequently, the m.593T > C mutation-induced deficiencies may dysregulate the expression of nucleus-encoding mitochondrial proteins involved in mitochondrial biogenesis, autophagy, and apoptosis (32). To further investigate the molecular mechanism of m.593T > C mutation, cybrid cell lines were constructed by transferring mitochondria from lymphoblastoid cell lines derived from an affected matrilineal relative in a Chinese family bearing the m.593T > C mutation and from a control individual lacking the mtDNA mutation into human mtDNA-less (*ρ*°) cells (33, 34).

**Figure 1:**
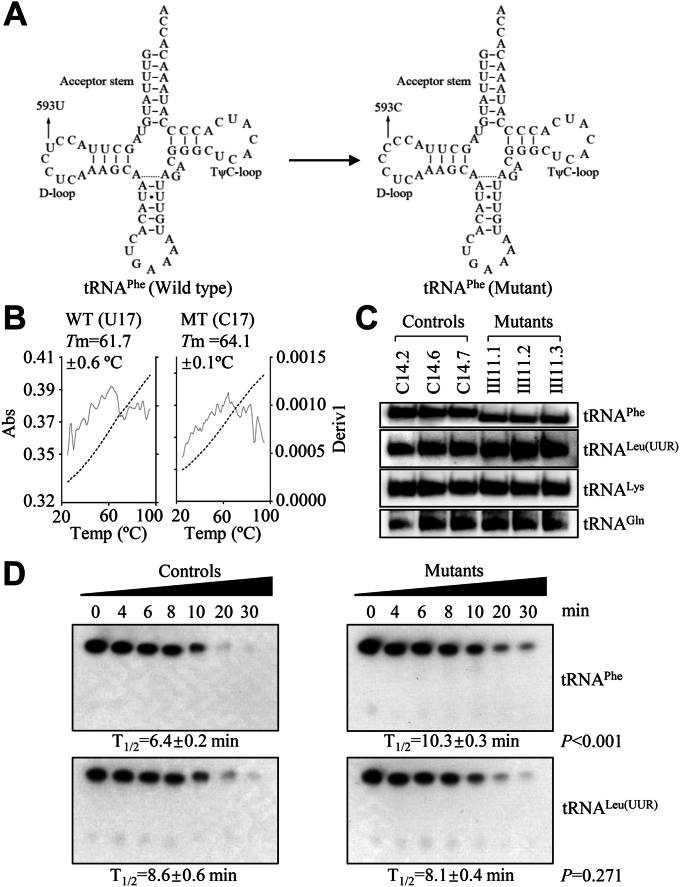
**Analysis for the conformation and stability of tRNA**^**Phe**^**.***A*, cloverleaf structure of human mitochondrial tRNA^Phe^. An *arrow* indicated the location of the m.593T > C mutation. *B*, thermal stability of wild type (U17) and mutant (C17) tRNA^Phe^ transcripts. Melting profiles of WT and MT tRNA^Phe^ transcripts were measured at 260 nm with a heating rate of 1 °C/min from 25 to 95 °C (*dotted lines*). First derivative (d*A*/d*T*) against temperature curves were shown to highlight the *T*m value transitions (*solid lines*). The means and standard deviations of *T*m were based on three independent determinations. *C*, assessment of conformation changes by PAGE analysis of tRNA^Phe^ under native conditions. Two micrograms of total mitochondrial RNA from various cell lines were electrophoresed through polyacrylamide gel, electroblotted, and hybridized with DIG-labeled oligonucleotide tRNA probes for tRNA^Phe^, tRNA^leu(UUR)^, tRNA^Lys^ and tRNA^Gln^ under native condition. Three independent determinations were performed. *D*, S1 digestion patterns of tRNA^Phe^ and tRNA^Leu(UUR)^, purified from control and mutant cybrids. Two micrograms of RNAs were used for the S1 cleavage reaction at various lengths (from 0 to 30 min). Cleavage products of tRNAs were resolved in 10% desaturating PAGE gels with 8 M urea, electroblotted, and hybridized with 3′ end DIG-labeled oligonucleotide probes specific for tRNA^Phe^ and tRNA^Leu(UUR)^, respectively. The graph shows the results of a representative experiment among three independent experiments.

These cybrid lines were analyzed for the effects of the m.593T > C mutation on mitochondrial translation, OXPHOS, mitochondrial membrane potential, and production of reactive oxidative species (ROS). These cell lines were further assessed for the impact of m.593T > C mutation on mitochondrial dynamics, autophagy, and apoptosis.

## Results

### Abnormal stability and conformation of tRNA^Phe^

To examine the effect of m.593T > C mutation on the stability of tRNA^Phe^, we measured the melting temperatures (*T*m) of wild-type (WT) and mutant (MT) tRNA^Phe^ transcripts by calculating the derivatives of absorbance against a temperature curve. As shown in Figure 1*B*, the *T*m values of WT (U17) and MT (C17) transcripts were 61.7 ± 0.6 °C and 64.1 ± 0.1° C（*p* = 0.003), respectively, indicating that the m.593T > C mutation affected the stability of tRNA^Phe^.

To assess whether the m.593T > C mutation affected the conformation of tRNA^Phe^, total mitochondrial RNAs from mutant and control cybrids were electrophoresed through a 10% polyacrylamide gel (native condition) in Tris borate-EDTA buffer and then electroblotted onto a positively charged nylon membrane for hybridization analysis with oligodeoxynucleotide probes for tRNA^Phe^, tRNA^Leu(UUR)^, tRNA^Lys^, and tRNA^Gln^, respectively. As shown in Figure 1*C*, electrophoretic patterns revealed that the tRNA^Phe^ in three mutant cybrids carrying the m.593T > C mutation migrated faster than those of three control cybrids lacking this mutation.

We further evaluated if the m.593T > C mutation perturbed the structures of tRNAs by analyzing the sensitivity of tRNA^Phe^ and tRNA^Leu(UUR)^ from control and mutant cell lines to digestion with the nuclease S1. The resultantly digested products from control and mutant cell lines were then followed by Northern blot analysis using tRNA probes that hybridized only to 3′ half tRNAs. As illustrated in Figure 1*D*, the tRNA^Phe^ from mutant cell lines were less sensitive to S1-mediated digestion than those from control cell lines. Conversely, there was no significant difference between the sensitivity of tRNA^Leu(UUR)^ from control and mutant cell lines to digestion with the nuclease S1. These data validated that the m.593T > C changed the conformation of mitochondrial tRNA^Phe^.

To investigate whether there was an effect of m.593T > C mutation on the half-life of tRNA^Phe^, we treated the various cell lines with ethidium bromide (EtBr) (250 ng/ml) to block mitochondrial RNA synthesis and extracted total cellular RNA at various time points after the addition of the drug. We then subjected mitochondrial RNAs from various cell lines to Northern blot analysis and hybridized them with digoxigenin (DIG)-labeled oligodeoxynucleotide probes specific for tRNA^phe^, tRNA^Leu(UUR)^, and 5S rRNA as a loading control (Fig. S1*A*). For comparison, tRNA half-life was calculated through the best least-squares line fit. There were no significant differences in the half-lives of tRNA^Phe^ and tRNA^Leu(UUR)^ between mutant and control cell lines (Fig. S1*B*). This suggested that m.593T > C mutation did not have a significant effect on the half-life of tRNA^Phe^.

To evaluate if the m.593T > C mutation affected the aminoacylation of tRNA^Phe^, we examined the aminoacylation properties of tRNA^Phe^, tRNA^Thr^, and tRNA^Leu(UUR)^ by the use of electrophoresis in an acidic urea PAGE system to separate uncharged tRNA species from the corresponding charged tRNA, electroblotting and hybridizing with the tRNA probes as described above. As shown in Fig. S2*A*, the upper and lower bands represented the charged and uncharged tRNA, respectively. To further distinguish nonaminoacylated tRNA from aminoacylated tRNA, samples of tRNAs were deacylated by being heated for 10 min at 60 °C at pH 8.3 and then run in parallel. Only one band (uncharged tRNA) was present in both mutant and control cell lines after deacylating. As shown in Fig. S2, there were no obvious differences in the electrophoretic mobility and levels of tRNA^Phe^, tRNA^Leu(UUR),^ and tRNA^Thr^ between mutant and control cell lines.

### Reduced levels of mtDNA encoding proteins

**Figure 2:**
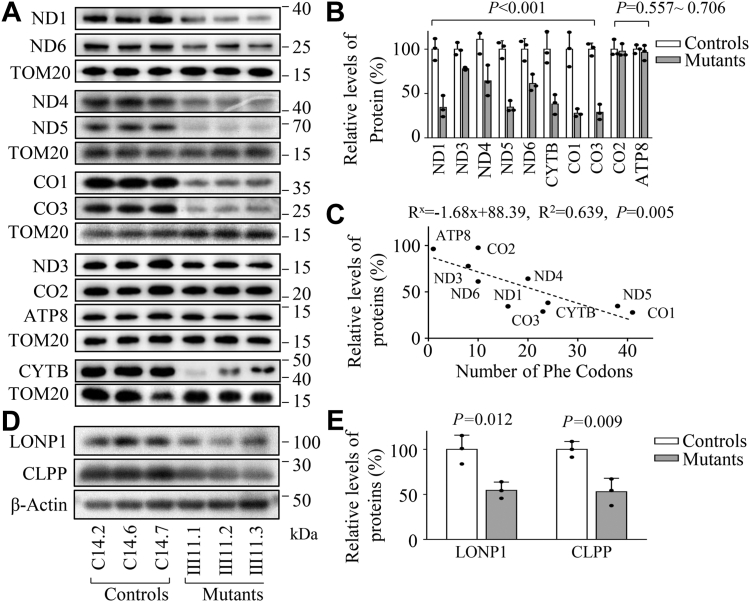
**Western blotting analysis of mitochondrial proteins.***A*, 20 micrograms of total cellular proteins from various cell lines were electrophoresed through a denaturing polyacrylamide gel, electroblotted, and hybridized with antibodies for 10 subunits of OXPHOS encoded by mtDNA and with TOM20 as a loading control, respectively. *B*, quantification of mitochondrial proteins. The average relative each polypeptide content per cell was normalized to the average content per cell of TOM20 in each cell line. The values for the latter are expressed as percentages of the average values for the control cybrids. The calculations were based on three independent determinations. The error bars indicate two standard deviations (SD) of the means. *P* indicates the significance, according to the *t* test, of the differences between mutant and control cell lines. *C*, relationship between average relative levels of the 10 polypeptides in the mutant cell lines and the number of phenylalanine codons. Lines of best fit (least squares method) are shown, R^2^ = 0.6141. The curve shown describes the equation: R^x^ = −1.5008x + 84.11, whose parameters have been optimized to make the best fit to the data. R^x^ is the level of labeling of a polypeptide having x number of Phe residues in mutant cell lines relative to the level in control cell lines. *D*, western blot analysis of CLPP involved in mitochondrial ribosome assembly and LONP1 that is a nuclear-encoded mitochondrial protease. *E*, quantification of the levels of CLPP and LONP1 in mutant and control cell lines as described above.

Previous investigations using lymphoblastoid cell lines revealed various reductions of six mtDNA-encoding proteins (30). To further assess if the m.593T > C mutation impaired mitochondrial translation, Western blot analysis was carried out to examine the levels of 10 mtDNA encoding polypeptides in mutant and control cell lines using TOM20 as a loading control. As shown in Figure 2*A*, the levels of ND1, ND3, ND4, ND5, and ND6 (subunits 1, 3, 4, five and six of NADH:ubiquinone oxidoreductase, complex I), CYTB (cytochrome b of ubiquinone cytochrome c oxidoreductase, complex III), CO1, and CO3 (subunits 1, three of cytochrome c oxidase, complex IV) exhibited variable reductions in the mutant cell lines, whereas the levels of CO2 (subunit II of cytochrome c oxidase) and ATP8 (subunits eight of the H^+^-ATPase) in the mutant cell lines were comparable with those of control cell lines. As shown in Figure 2*B*, the average levels of ND1, ND3, ND4, ND5, ND6, CYTB, CO1, CO2, CO3, and ATP8 in these mutant cells carrying 593T > C mutation were 34%，77%，64%, 34%, 61%，38%, 27%, 97%, 28%, and 96% of the average levels of control cells, respectively.

As shown in Figure 2*C* and Table S1, the experimentally determined levels of all polypeptides, when related to the number of phenylalanine codons in the corresponding mRNAs, conformed well (*p* = 0.005) to the equation R^x^ = −1.68x + 88.39, whose parameters have been optimized to make the best fit to the data. R^x^ is the level of labeling of a polypeptide having x number of Phe residues in the mutant cybrids relative to the level in the control cybrids. However, the reductions in levels of various polypeptides did not vary in relationship to the proportion of phenylalanine codons in the corresponding mRNAs (Table S1).

We then examined the levels of five subunits of the phosphorylation system (OXPHOS) in control and mutant cell lines by Western blot analysis. As shown in Fig. S3, *A* and *B*, the average levels of mtDNA-encoded CO2, and other four polypeptides (NDUFB8 of NADH:ubiquinone oxidoreductase; SDHB of succinate ubiquinone oxidoreductase; UQCRC2 of ubiquinol cytochrome c reductase and ATP5A of H^+^-ATPase), encoded by nuclear genes, in mutant cell lines were comparable with those in control cell lines.

To test whether the m.593T > C mutation affected the mitochondrial proteostasis, we examined the levels of CLPP involved in mitochondrial ribosome assembly (35) and LONP1 which is a nuclear-encoded mitochondrial protease crucial for organelle homeostasis (36) in mutant and control cells. As shown in Figure 2, *D* and *E*, the levels of CLPP and LONP1 in the mutant cells were 52% and 54% of those in the control cells, respectively. These indicated that m.593T > C mutation impaired mitochondrial proteostasis.

### Decreases in the stability and activities of OXPHOS complexes

**Figure 3:**
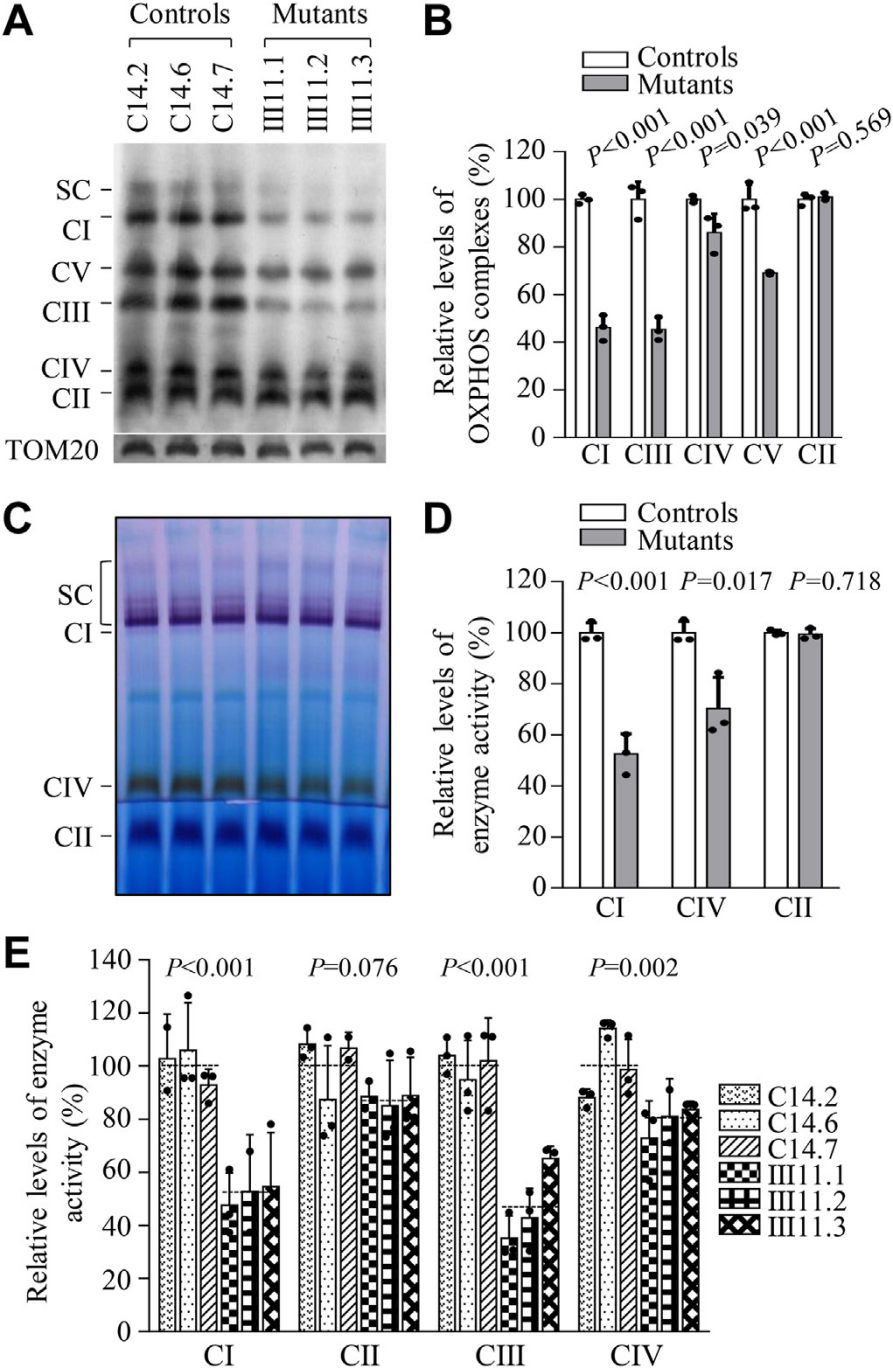
**Analysis of OXPHOS complexes.***A*, the steady-state levels of five OXPHOS complexes by Blue-Native gel electrophoresis. 30 micrograms of mitochondrial proteins from mutant and control cell lines were electrophoresed through BN gel, electroblotted, and hybridized with an antibody cocktail specific for subunits of each OXPHOS complex as well as TOM20 as a loading control. *B*, quantification in the levels of complexes I, II, III, IV, and V in mutant and control cell lines. The calculations were based on three independent experiments. *C*, in-gel activity of complexes I, II, and IV. 20 micrograms of mitochondrial proteins from various cell lines were used for BN-PAGE, and the activities of complexes were measured in the presence of specific substrates (NADH and NTB for complex I, sodium succinate, phenazine methosulfate, and NTB for complex II, and DAB and cytochrome c for complex IV). *D*, quantification of in-gel activities of complexes I, II and IV. The calculations were based on three independent determinations in each cell line. *E*, enzymatic activities of respiratory chain complexes. The activities of respiratory complexes were investigated by enzymatic assay on complexes I, II, III and IV in mitochondria isolated from various cell lines. The calculations were based on four independent determinations in each cell line. Graph details and symbols are explained in the legend to Figure 2.

We examined the consequence of m.593T > C mutation on the assembly and activity of OXPHOS complexes. Mitochondria isolated from various cell lines were separated by blue native polyacrylamide gel electrophoresis (BN-PAGE) and Western blot analysis (37, 38). As illustrated in Figure 3*A*, the mutant cybrids bearing the m.593T > C mutation revealed aberrant assembly of complex I, III, IV, and V, respectively. As shown in Figure 3*B*, the average levels of complex I (CI), complex III (CIII), complex IV (CIV), and complex V (CV) in three mutant cybrids were 46%, 45%, 86%, and 69% of those in three control cybrids after normalization to TOM20, respectively. However, the levels of complex II (CII) in mutant cybrids were comparable with those in control cybirds. The lower levels of the respiratory complexes I, III, IV, and V may be due to the misfolded and/or misassembled of these complexes (39).

We then analyzed the stability and activities of complexes I, II, and IV using the in-gel activity assay. Mitochondrial membrane proteins isolated from mutant and control cybrids were separated by BN-PAGE and stained with specific substrates of complexes I, II, and IV (38). Defective assembly of complex I and IV was further confirmed in mutant cybrids harboring the m.593T > C mutation, as compared with those in control cybrids (Fig. 3, *C* and *D*). In particular, the average in-gel activities of complex I and IV of three mutant cybrids were 52% and 70%, relative to the average values of three control cybrids, respectively. In contrast, there was no significant difference in the average in-gel activities of complex II between mutant and control cybrids.

To further evaluate the effect of m.593T > C mutation on oxidative phosphorylation, we measured the activities of OXPHOS complexes by the use of isolating mitochondria from mutant and control cybrids. The activity of complex I (NADH ubiquinone oxidoreductase) was determined through the oxidation of NADH with ubiquinone as the electron acceptor (40). The activity of complex II (succinate ubiquinone oxidoreductase) was examined by the artificial electron acceptor DCPIP. The activity of complex III (ubiquinone cytochrome c oxidoreductase) was measured through the reduction of cytochrome c by using D-ubiquinol-2 as the electron donor. The activity of complex IV (cytochrome c oxidase) was monitored through the oxidation of cytochrome c. As shown in Figure 3*E*, the average activities of complexes I, III, and IV in three mutant cybrids were 51%, 48%, and 79% of the mean values measured in three control cybrids, respectively, while the average activity of complex II in three mutant cybrids was comparable with those in three control cybrids.

### Decreased mitochondrial membrane potential

**Figure 4:**
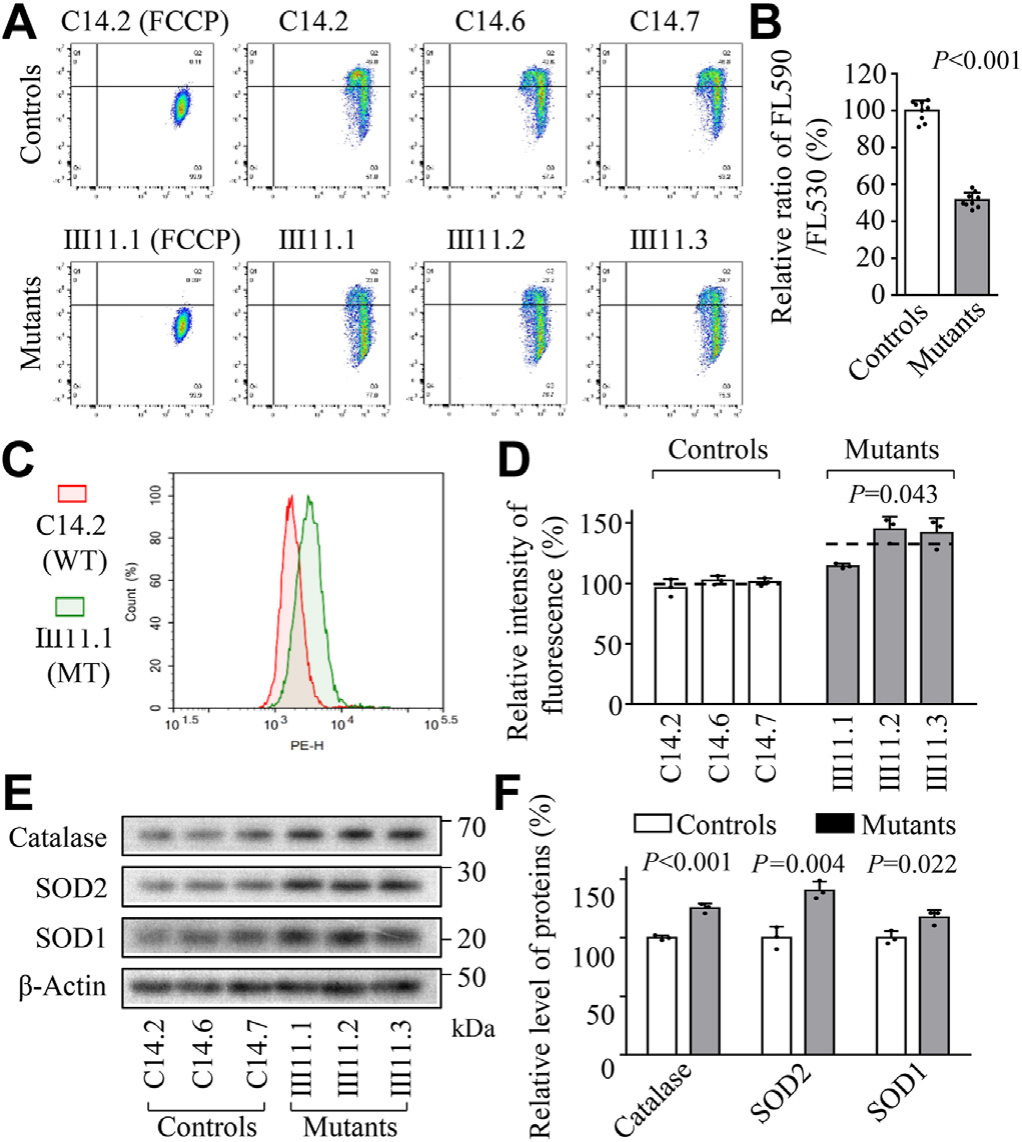
**Mitochondrial membrane potential and ROS production analysis.***A*, mitochondrial membrane potential analysis. Represented flow cytometry images of mutant and control cell lines with and without 10 μM of FCCP. *B*, the relative ratios of JC-10 fluorescence intensities at excitation/emission of 490/530 nm and 490/590 nm. *C*, the rates of ROS generation by mitochondria in living cells from three mutants and three control cybrids were analyzed by a Novocyte flow cytometer (ACEA Biosciences) using the mitochondrial superoxide indicator MitoSOX-Red (5 mM). Flow cytometry histogram showing MitoSOX-Red fluorescence of various cell lines. *D*, the relative ratios of intensity were calculated. The average value of three independent determinations for each cell line is shown. *E*, Western blotting analysis of anti-oxidative enzymes SOD1, SOD2, and catalase in six cell lines with β-Actin as a loading control. *F*, quantification of SOD1, SOD2 and catalase. Three independent experiments were made for each cell line. Graph details and symbols are explained in the legend to Figure 2.

The mitochondrial membrane potential (Δ*Ψ*_m_) generated by complexes I, III, and IV is an essential component in the process of energy storage during oxidative phosphorylation (41, 42). The ΔΨ_m_ of mutant and control cybrids were examined through the fluorescence probe JC-10 assay system. The ratios of fluorescence intensities Ex/Em = 490/590 and 490/530 nm (FL590/FL530) were recorded to delineate the ΔΨ_m_ of each sample. The relative ratios of FL590/FL530 geometric mean between mutant and control cybrids were calculated to represent the level of ΔΨ_m_, as described elsewhere (42). As illustrated in Figure 4, *A* and *B*, the average ΔΨ_m_ of three mutant cybrids was 51.5% of the mean value measured in three control cybrids, whereas the levels of ΔΨ_m_ in mutant cell lines in the presence of FCCP were comparable to those of control cell lines.

### Enhanced the production of mitochondrial ROS

The levels of mitochondrial ROS among these cells were determined using a MitoSOX assay *via* flow cytometry (43). Geometric mean intensity was recorded to measure and delineate the rate of ROS of each sample. As shown in Figure 4, *C* and *D*, the levels of ROS generation in the mutant cybrids ranged from 114.3% to 144.8%, with an average of 133.6% of the mean values measured in the control cybrids under unstimulated conditions.

To further assess if the m.593T > C mutation-induced mitochondrial ROS production affected the antioxidant systems, we examined the levels of three antioxidant enzymes: SOD2 in mitochondria, and SOD1 and catalase in the cytosol in the various cell lines (44, 45). As shown in Figure 4, *E* and *F*, the mutant cell lines revealed marked increases in the levels of SOD2 (125.2%), SOD1 (140.2%), and catalase (117.4%), respectively.

### Imbalanced mitochondrial dynamics

**Figure 5:**
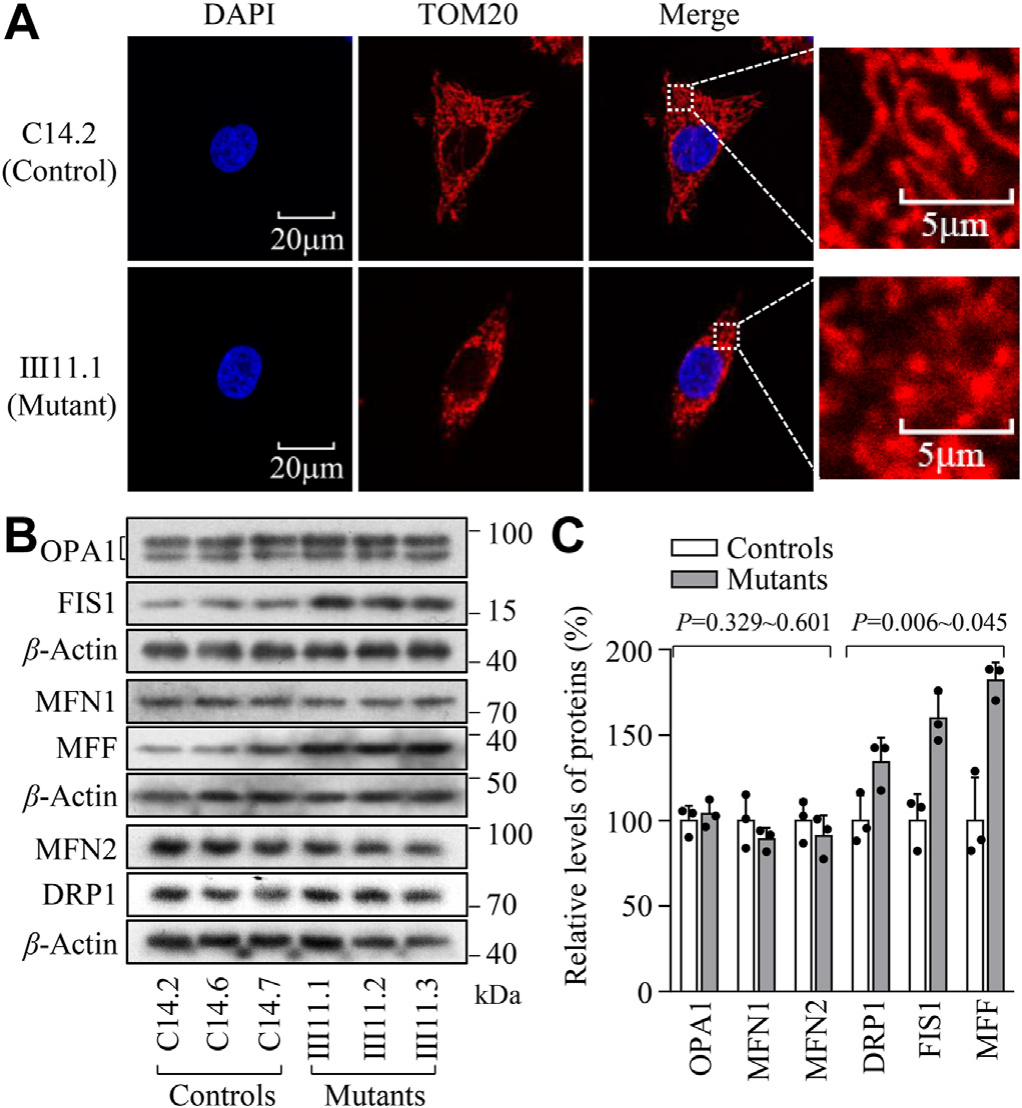
**Mitochondrial dynamics analysis.***A*, mitochondrial morphologies in mutant (Ⅲ11.1) and control (C14.2) cell lines were visualized by immunofluorescent labeling with TOM20 antibody conjugated to Alex Fluor 594 (*red*) and analyzed by confocal microscopy. *B*, Western blot analysis for mitochondrial dynamics-related proteins. 20 micrograms of total cellular proteins from various cell lines were electrophoresed, electroblotted, and hybridized with mitochondrial fusion-related proteins OPA1, MFN1, and MFN2, and mitochondrial fission-related proteins DRP1, FIS1 and MFF. *C*, quantification of mitochondrial dynamics proteins in the mutant cell lines and control cell lines. Graph details and symbols are explained in the legend to Figure 2.

To evaluate if the m.593T > C mutation affected the mitochondrial dynamics, we examined the mitochondrial fission and fusion of mutant and control cybrids by immunofluorescence and Western blot analyses. As shown in Figure 5*A*, the immunofluorescence patterns of labeled cells with mouse monoclonal antibody to TOM20 revealed highly fragmented mitochondria in the mutant cells, compared with those in the control cells. The levels of three fusion-related proteins (MFN1, MFN2, and OPA1) and three fission-related proteins (DRP1, FIS1, and MFF) in mutant and control cell lines were further assessed by Western blot analysis (46, 47). The average levels of DRP1, FIS1, and MFF in three mutant cell lines were 134.1%, 159.6%, and 182.1% of the average values measured in three control cell lines, respectively. However, the average levels of MFN1, MFN2, and OPA1 in mutant cell lines were comparable with those in control cell lines. These data indicated that the m.593T > C mutation promoted the mitochondrial fission process in the mutant cybrids.

### Autophagy is a pathological increase in cells in patients with deafness

**Figure 6:**
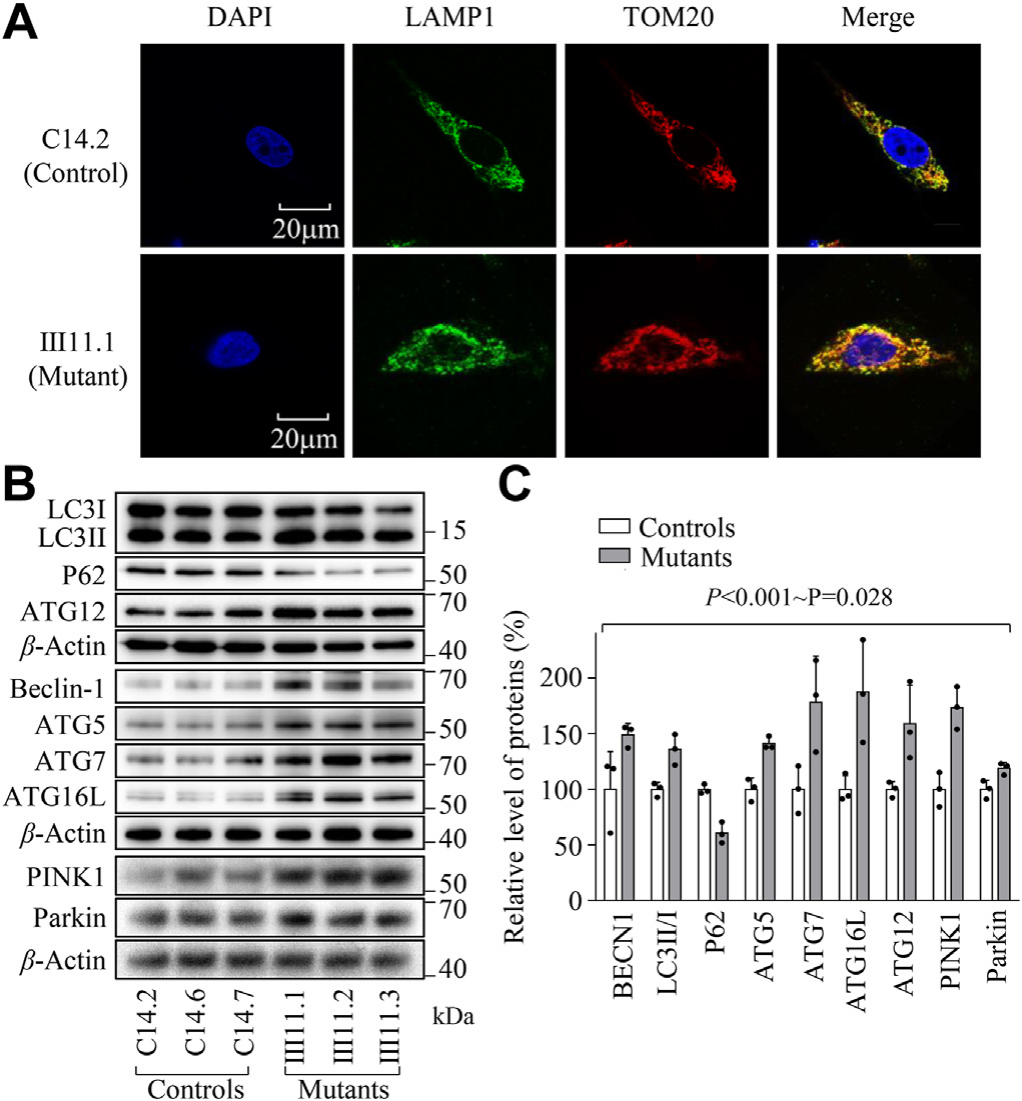
**Assessment of autophagy.***A*, the distributions of LAMP1 from mutant (Ⅲ11.1) and control (C14.2) cell lines were visualized by immunofluorescent labeling with TOM20 antibody conjugated to Alex Fluor 594 (*red*) and LAMP1 antibody conjugated to Alex Fluor 488 (*green*) analyzed by confocal microscopy. DAPI-stained nuclei were shown by the blue fluorescence. *B*, Western blot analysis for autophagic related protein. 20 micrograms of total cellular proteins from various cell lines were electrophoresed, electroblotted, and hybridized with BECN1, ATG5, ATG7, ATG16L, ATG12, LC3, P62, PINK1, and Parkin antibodies and with β-Actin as a loading control. *C*, quantification of autophagy-related proteins in the mutant cell lines and control cell lines. Graph details and symbols are explained in the legend to Figure 2.

An imbalance of mitochondrial dynamics due to mitochondrial dysfunctions may facilitate autophagy (34). The autophagy states of three mutant and three control cell lines were analyzed using immunoblotting and immunofluorescence assays. As shown in Figure 6*A*, mutant cell lines displayed markedly increased levels of LAMP1 (lysosome-associated membrane glycoprotein1), as compared with those in control cybrids, indicating that the m.593T > C mutation altered the autophagy process. As shown in Figure 6*B*, the effects of m.593T > C mutation on autophagy were further assessed by Western blot analysis using five antibodies of autophagy-related proteins [ATG5, ATG7, ATG16L, ATG12 (autophagy-related 5, 7, 16L and 12), and BECN1 (beclin 1)] and mitophagy-related proteins [LC3 (microtubule-associated protein 1A/1B light chain 3), P62 (sequestosome 1)] (48). Indeed, LC3, P62, and Beclin-1 are involved in the initiation phase of autophagy, in mutant and control cell lines. The activity of the conserved Atg12–Atg5-Atg16 complex is essential for autophagosome formation and membrane elongation, while ATG7 plays a central role in autophagosome biogenesis by conjugating ATG5 to ATG12 (47). As shown in Figure 6*C*, the average levels of ATG5, ATG7, ATG16L, ATG12, BECN1, and LC3II/I in mutant cybrids were 148.8%, 141.0%, 178.0%, 187.4%, 158.9%, and 135.8% of the mean values measured in control cybrids, respectively. As an additional autophagy marker, we used the protein SQSTM1/p62, which is inversely correlated with autophagy activity (48). By contrast, the average levels of P62 in mutant cybrids were 60.7% of the mean values measured in control cybrids. These results implicated that the m.593T > C mutation promoted autophagosome formation and maturation.

Mitophagy is a specific form of autophagy that selectively removes damaged mitochondria by autophagosomes and their subsequent catabolism by lysosomes (49). Upon acute mitochondrial dysfunction, the PINK1-Parkin pathway is activated by Parkin recruitment from the cytosol to the mitochondrial surface, ultimately leading to mitophagy (50). As shown in Figure 6, *A* and *B*, the average levels of PINK1 and Parkin in mutant cybrids were 173.3% and 119.0% of the mean values measured in control cybrids, respectively. These data indicated that the m.593T > C mutation promoted mitophagy in mutant cybrids.

### Promoting intrinsic apoptosis

**Figure 7:**
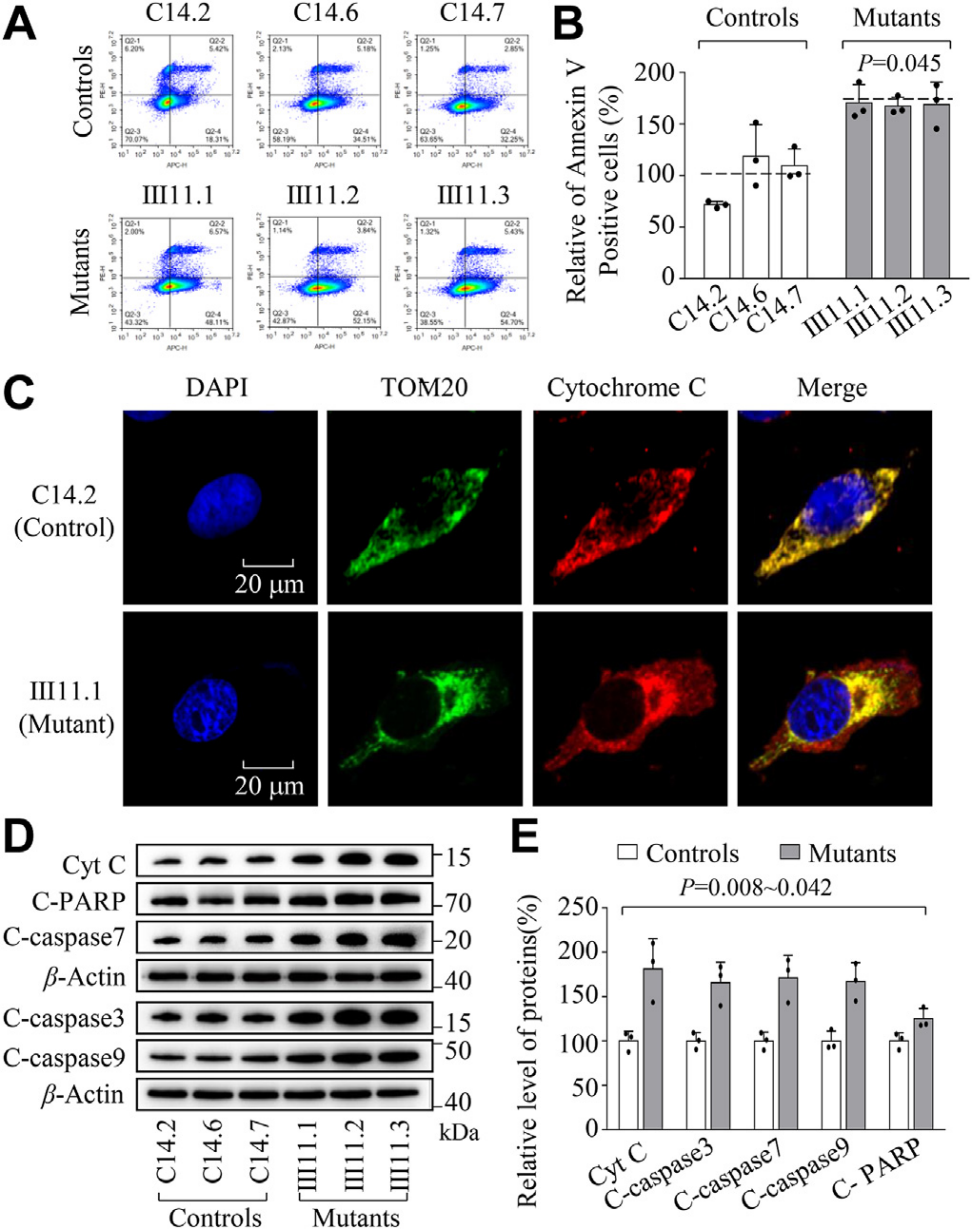
**Apoptosis assays.***A*, annexin V/PI apoptosis assay by flow cytometry. Cells were harvested and stained with Annexin V and 1 μl of propidium iodide. The percentage of Annexin V-positive cells were then assessed. *B*, relative Annexin V-positive cells from various cell lines. Three independent determinations were done in each cell line. *C*, immunofluorescence analysis. The distributions of cytochrome c from mutant Ⅲ11.1 and control C14.2 cybrids were visualized by immunofluorescent labeling with TOM20 antibody conjugated to Alex Fluor 488 (*green*) and cytochrome c antibody conjugated to Alex Fluor 594 (*red*) analyzed by confocal microscopy. DAPI-stained nuclei were identified by their blue fluorescence. *D*, Western blotting analysis. Cellular proteins (20 μg) from various cell lines were electrophoresed, electroblotted and hybridized with several apoptosis-associated protein antibodies: Cyt C, C-PARP, caspases 3, caspases seven and caspases 9 with β-Actin as a loading control. *E*, quantification of apoptosis-associated proteins. Three independent experiments were made for each cell line. Graph details and symbols are explained in the legend to Figure 2.

Mitochondrial fission machinery actively participates in the process of intrinsic apoptosis (51). The impact of m.593T > C mutation on the apoptotic process was examined by Annexin V/PI-based flow cytometry for cellular apoptosis, immunocytostaining assays and Western blot analysis. As shown in Figure 7, *A* and *B*, the average ratio of Annexin V-positive cells in the mutant cybrids carrying the m.593T > C mutation was 168% of the mean values measured in the control cybrids. As shown in Figure 7*C*, the immunofluorescence patterns of cybrids double labeled with mouse monoclonal antibody specific for the cytochrome c and rabbit monoclonal antibody to TOM20 exhibited markedly increased levels of cytochrome c, as compared with the control cybrids. The levels of cytochrome c in cytosol in mutant and control cybrids were further assessed by Western blot analysis. As shown in Figure 7, *C* and *D*, the average levels of cytochrome c in mutant cybrids were 181.2% (*p* = 0.001), relative to the average values in control cybrids. We then measured the levels of apoptosis-related proteins: cleaved PARP, caspases 3, 7, and 9 in mutant and control cell lines using Western blot analysis. As shown in Figure 7, *D* and *E*, the average levels of cleaved PARP, caspases 3, 7, and 9 in three mutant cell lines were 124%, 165%, 171%, and 166%, of the average values measured in the three control cell lines, respectively.

## Discussion

In the present study, we have further investigated the molecular pathogenesis of deafness-associated tRNA^Phe^ 593T > C mutation. The m.593T > C mutation changed a highly conserved uracil to cytosine at position 17 of DHU-loop, where the position is important for the structure and function of tRNA (4, 31). Therefore, it was hypothesized that m.593T > C mutation perturbed the structure and function of tRNA^Phe^. In this study, we demonstrated that the m.593T > C mutation increased structural stability of tRNA^Phe^ molecule, as evidenced by the fact that the *T*m in tRNA^Phe^ with C17 was 2.4^o^C higher than the same tRNA with U17. The greater stability of mutant tRNA^Phe^ molecule was further supported by resistance to S1-mediated digestion in mutant tRNA than WT molecules. Furthermore, the m.593T > C mutation caused the conformation change of tRNA^Phe^, as suggested by faster electrophoretic mobility of mutated tRNA with respect to the WT molecule *ex vivo*, consistent with the conformation changes in tRNA^Met^ carrying the 4435A > G and tRNA^His^ bearing the 12201T > C mutations (42, 44). However, the m.593T > C mutation did not have significant effects on aminoacylation and the half-life of tRNA^Phe^, in contrast to the observations that m.5783C > T mutation at the TΨC stem of tRNA^Cys^ and m.12201T > C mutation at the acceptor stem of tRNA^His^ (27, 44). This abnormal stability and conformation of mutant tRNA molecule accounted for significantly reduced levels of tRNA^Phe^ in the mutant cell lines carrying the m.593T > C mutation (30, 52).

The m.593T > C mutation-induced alterations in tRNA metabolism led to impairment of mitochondrial translation and subsequently deficient oxidative phosphorylation (30, 52). In this study, variable decreases (an average decrease of ∼45%) in 10 mtDNA-encoded polypeptides were observed in the mutant cybrids carrying the m.593T > C mutation, comparable effects were seen in cells bearing the m.4435A > G or m.7516delA mutation (22, 42). Strikingly, mutant cybrids carrying the m.593T > C mutation revealed marked reductions (62% to 72%) in the levels of five polypeptides (ND1, ND5, CO1, CO3, and CYTB) harboring higher numbers of phenylalanine. By contrast, the levels of CO2 and ATP8 with lower numbers of phenylalanine codons in mutant cybrids were comparable with those in control cybrids. As shown in the Table S1, the reduced levels of these polypeptides in mutant cybrids were significantly correlated with the numbers of phenylalanine, in agreement with previous study in cells carrying the tRNA^Lys^ 8344A > G and tRNA^Cys^ 5783C > T mutations (27, 53). Alternatively, decreased protein levels were contributed by impaired mitochondrial proteostasis, supported by significantly reduced levels of CLPP and LONP1 (35, 36, 54).

The m.593T > C mutation-induced reductions of mtDNA encoding subunits of OXPHOS resulted in aberrant assembly and stability of complexes I, III, and IV as well as intact supercomplexes observed in the mutant cell lines carrying m.593T > C mutation. In fact, human OXPHOS complex is comprised of 12 mtDNA-encoded subunit(s) and 80 nuclear-encoded subunits, except complex II (55). As a consequence, these defects gave rise to the reduced activities of OXPHOS complexes. In particular, the impaired synthesis of ND1, ND5, CYTB, and CO1, CO3 was responsible for the decreased activities of complexes I, III, and IV, respectively. The resultant respiratory deficiencies diminished mitochondrial ATP production and membrane potential (30). The impairment of both OXPHOS and membrane potential increased the production of ROS and expression of SOD2, SOD1, and catalase in mutant cell lines (44, 45). The elevated production of ROS can increase a vicious cycle of oxidative stress in the mitochondria, thereby worsening the damage to mitochondrial and cellular proteins, lipids, and nuclear acids (45).

The m.593T > C mutation-induced dysfunction of OXPHOS and overproduction of ROS may make mitochondria undergo constant fission to repair damaged OXPHOS components, which allows segregation of damaged mitochondria *via* the fission process, exchange of materials between healthy mitochondria *via* the fusion process, and finally the elimination of damaged mitochondria *via* mitophagy (56). Here, mutant cells bearing the m.593T > C mutation exhibited an imbalance of mitochondrial dynamics including abnormal morphology of mitochondria such as markedly increased fragments and dysregulation of mitochondrial dynamics-related proteins. The mutant cells carrying the m.593T > C mutation revealed markedly increased expression of nucleus-encoding fission-related genes *DRP1*, *FIS1*, and *MFF* than these control cells lacking the mutation. By contrast, the levels of three fusion-related proteins (OPA1, MFN1, and MFN2) in mutant cells harboring the m.593T > C mutation were comparable with those in the control cells. These strongly indicated that the m.593T > C mutation regulated mitochondrial dynamics by promoting fission.

Imbalance of mitochondrial dynamics and impaired membrane potentials by the m.593T > C mutation modulated mitophagy which is a mitochondria-specific type of autophagy to dispose of damaged mitochondria (50, 57). In this study, the cells bearing the m.593T > C mutation displayed marked increases in the levels of LAMP1 and LC3 (58). The m.593T > C mutation affected the process of autophagy, including the initiation phase, supported by increased levels of Beclin-1 but decreased levels of P62, formation and maturation of autophagosome, evidenced by raising levels of ATG5, ATG7, ATG16L1 and ATG12-ATG5 in the cells carrying the m. 593T > C mutation (50, 56, 58). The m.593T > C mutation-induced autophagy dysregulation was consistent with increased autophagy in the cells carrying the tRNA^Met^ 4435A > G, tRNA^Ile^ 4295A > G and tRNA^Ala^ 5587C > T mutations (26, 32, 59), suggesting that mitochondrial tRNA mutations upregulated autophagy pathway. Furthermore, the m.593T > C mutation upregulated the PARKIN-dependent mitophagy, evidenced by markedly increased levels of PARKIN and PINK1 in cells carrying the m.593T > C mutation, in contrast with reduced levels in PARKIN and PINK1 in the cells ND1 3460G > A or ND6 14484T > C mutation (60, 61). These suggested that the m.593T > C mutation upregulated mitophagy, especially the ubiquitination-dependent pathway.

The m.593T > C mutation-induced elevation of mitochondrial fission and ROS production and diminished membrane potential promoted the intrinsic apoptotic process for the removal of damaged cells. In this study, we showed that the m.593T > C mutation conferred defects in the apoptosis, evidenced by 68% increased levels of Annexin V intensity. The impaired apoptosis was further evidenced by raising levels of cytochrome *c* in the mutant cybrids. The increasing release of cytochrome *c* promoted the activation of PARP, caspase 3, 7, and 9, which subsequently initiates cell death (51, 62). These m.593T > C mutation-induced alterations may lead to damaged or deficient inner ear hair cells that are particularly vulnerable to neurodegeneration related to oxidative phosphorylation, thereby contributing to the development of hearing loss (63, 64). The hearing-specific phenotypes of this tRNA mutation may be attributed to the tissue-specificity of OXPHOS *via* tRNA metabolism or modulate intracellular signaling related to mitochondrial and cellular integrity (65, 66, 67, 68, 69).

In summary, we demonstrated the impact of deafness-associated tRNA^Phe^ 593T > C mutation on the mitochondrial and cellular integrity contributing to the pathological process of hearing loss. The m.593T > C mutation has structural and functional consequences of tRNA^Phe^. The aberrant tRNA metabolism resulted in impairment of mitochondrial protein synthesis, especially for those polypeptides with high phenylalanine codons, and subsequently deficient oxidative phosphorylation necessary for hearing function. These mitochondrial dysfunctions led to the mitochondrial dynamic imbalance towards fission, elevated mitophagy, and finally promoted intrinsic apoptosis. Our findings provide new insights into the pathophysiology of maternally inherited deafness arising from tRNA mutation-induced defects in mitochondrial and cellular integrity.

## Experimental procedures

### Cell lines and culture conditions

Immortalized lymphoblastoid cell lines were generated from one affected matrilineal relative (III-11, female, 45 years) of a Chinese family carrying the m.593T > C mutation and one genetically unrelated Chinese control individual (C14, female, 42 years) belonging to the same mtDNA haplogroup (G2a2a) lacking the mutation (Table S2) (30). Lymphoblastoid cell lines were grown in RPMI 1640 medium (Corning), supplemented with 10% fetal bovine serum. The 143B.TK^–^ cell line was grown in DMEM (containing 4.5 mg of glucose and 0.11 mg pyruvate per ml), supplemented with 100 μg of BrdU per ml and 5% FBS. The mtDNA-less ρ^o^206 cell line, derived from 143B.TK^–^ was grown under the same conditions as the parental line, except for the addition of 50 μg of uridine/ml (33). Transformation by cytoplasts of mtDNA-less ρ^o^206 cells was performed by using immortalized lymphoblastoid cell lines, as detailed previously (33, 34). The cybrids derived from each donor cell line were analyzed for the presence and level of the m.593T > C mutation and mtDNA copy numbers as detailed elsewhere (27). Three cybrids derived from each donor cell line with homoplasmy of mtDNA mutations and similar mtDNA copy numbers were used for the following biochemical characterization. All cybrid cell lines were maintained in the same medium as the 143B.TK^–^ cell line.

### UV melting assay

UV melting assays were carried out as described previously (20, 42). The plasmids bearing full-length WT and MT tRNA^Phe^ sequences used for *in vitro* transcription were construed as previously (70) and their sequences were confirmed by Sanger sequence analysis (Fig. S4). The WT and mutant tRNA^Phe^ transcripts were generated by *in vitro* transcription by T7 RNA polymerase according to previous protocols (70). The plasmids bearing full-length WT and MT tRNA^Phe^ were used as templates for *in vitro* transcription. These tRNA^Phe^ transcripts were diluted in 50 mM sodium phosphate buffer (pH 7.0), including 50 mM NaCl, 5 mM MgCl_2,_ and 0.1 mM EDTA. Absorbance against temperature melting curves was measured at 260 nm with a heating rate of 1 °C/min from 25 to 95 °C *via* an Agilent Cary 100 UV Spectrophotometer.

### Mitochondrial tRNA analysis

For the tRNA Northern blot analysis, total cellular RNAs were obtained using TOTALLY RNA kit (Ambion) from various cell lines (∼2.0 × 10^7^ cells), as described previously (71). Two micrograms of total mitochondrial RNAs were electrophoresed through a 10% polyacrylamide/7 M urea gel in Tris-borate-EDTA buffer (TBE) (after heating the sample at 65 °C for 10 min), and then electroblotted onto a positively charged nylon membrane (Roche) for the hybridization analysis with oligodeoxynucleotide probes. Oligodeoxynucleosides used for DIG-labeled probes of tRNA^Phe^, tRNA^Leu(UUR)^, tRNA^Lys^ and tRNA^Gln^, and 5S rRNA were as described elsewhere (22, 30, 42). DIG-labeled oligodeoxynucleotides were generated by using DIG oligonucleotide Tailing kit (Roche). The hybridization was carried out as detailed elsewhere (42).

The S1 nuclease cleavage analysis was performed as detailed elsewhere (20, 38, 42). In brief, 2 μg of total RNAs were incubated with 1 μg/μl total yeast tRNA and 1 U/μl S1 nuclease (Thermofisher) in the 5 μl reaction buffer containing 40 mM sodium acetate (pH 4.5), 300 mM NaCl and 2 mM ZnSO_4_. Reaction mixtures were incubated at 28 °C for indicated times and quenched by adding 5 μl loading buffer. Samples were electrophoresed through 10% denaturing polyacrylamide gel with 8 M urea and then electroblotted onto a positively charged nylon membrane for hybridization analysis with DIG-labeled oligodeoxynucleotide probes as described above.

Aminoacylation assays of tRNA^Phe^, tRNA^Leu(UUR),^ and tRNA^Thr^ from various cell lines were performed as detailed elsewhere (42). Quantification of density in each band was performed as detailed previously (42).

tRNA half-life measurements were performed as detailed elsewhere (27, 44). Briefly, various cell lines were incubated in a fresh medium containing 250 ng/ml of EtBr for the times indicated in the Figure 5*C*. Total cellular RNAs, extracted using TOTALLY RNA kit (Ambion) from various cell lines (∼1 × 10^7^ cells), were subjected to Northern blot analysis as detailed above.

### Western blot analysis

Western blotting analysis was carried out as detailed previously (23, 42). 20 micrograms of total proteins obtained from lysed mitochondria were denatured and loaded on sodium dodecyl sulfate (SDS) polyacrylamide gels. The gels were electroblotted onto a polyvinylidene difluoride (PVDF) membrane for hybridization. The antibodies used for this investigation were from Abcam [ND1 (ab74257), ND3 (ab170681), ND6 (ab81212), TOM20 (ab56783), Total OXPHOS Human WB Antibody Cocktail (ab110411) and P62 (ab56416) ], Proteintech Group [CYTB (55090-1-AP), CO2 (55070-1-AP), ATP8 (26723-1-AP), CLPP (15698-A-AP), LONP1 (15440-1-AP), OPA1 (27733-1-AP), FIS1 (10956-1-AP), MFN1 (13798-1-AP), MFF (17090-1-AP), MFN2 (12186-1-AP) and DRP1 (12957-1-AP)], ABclonal Technology [β-Actin (AC026), ND4 (A9941), ND5 (A17972), CO1 (A17889), CO3 (A17891), LC3 (A19665), Parkin (A0968) and Cyt C (A4912)], Cell Signaling [Autophagy Antibody Sampler Kit (4445) and Apoptosis Antibody Sampler Kit (9915)] and Abcepta [PINK1 (AW5456)]. Peroxidase AffiniPure goat anti-mouse IgG and goat anti-rabbit IgG (Jackson) were used as secondary antibodies and protein signals were detected using the ECL system (CW-BIO). Quantification of density in each band was performed as detailed elsewhere (42).

### Blue native gel electrophoresis analysis

BN-PAGE was performed by isolating mitochondrial proteins from mutant and control cell lines, as detailed elsewhere (37, 38). Samples containing 30 μg of total cellular proteins were separated on 3% to 12% Bis-Tris Native PAGE gel. The native gels were prewashed in cold water and then incubated with the substrates of complex I, complex II, and complex IV at room temperature as described elsewhere (38). After stopping the reaction with 10% acetic acid, gels were washed with water and scanned to visualize the activities of respiratory chain complexes.

### Enzymatic assays

The enzymatic activities of complexes I-IV were measured as detailed elsewhere (40).

### Assessment of mitochondrial membrane potential

Mitochondrial membrane potential was assessed with JC-10 Assay Kit-Microplate (Abcam) according to the general manufacturer's recommendations with some modifications, as detailed elsewhere (41, 42).

### ROS measurements

ROS measurements were conducted as detailed previously (43, 44).

### Annexin V/PI apoptosis assay by flow cytometry

For discrimination of apoptotic and non-apoptotic cells by Annexin V/PI staining. Cells were harvested and stained with Annexin V and 1 μl of propidium iodide (PI) (Themo Fisher Scientific) according to the manufacturer’s instruction. Each sample was detected by NovoCyte (ACEA Biosciences) and analyzed using NovoExpress software (59).

### Statistical analysis

Statistical analysis was carried out using the Student's unpaired, two-tailed *t* test contained in the Microsoft-Excel program. Unless indicated otherwise, a *p* value < 0.05 was considered statistically significant.

## Data availability

The authors declare that all relevant data of this study are available within the article or from the corresponding author (gminxin88@zju.edu.cn) upon reasonable request.

## Supporting information

This article contains supporting information.

## Conflict of interest

All authors declare that they have no conflicts of interest with contents of this article.
