# Supplementary material for: Deafness-associated tRNAPhe mutation impaired mitochondrial and cellular integrity
Source: J Biol Chem. 2024 Mar 27;300(5):107235. doi: 10.1016/j.jbc.2024.107235 (PMC11046301; doi:10.1016/j.jbc.2024.107235)
Supplement: Supporting Information [file mmc1.pdf]

Supplemental Figure S1

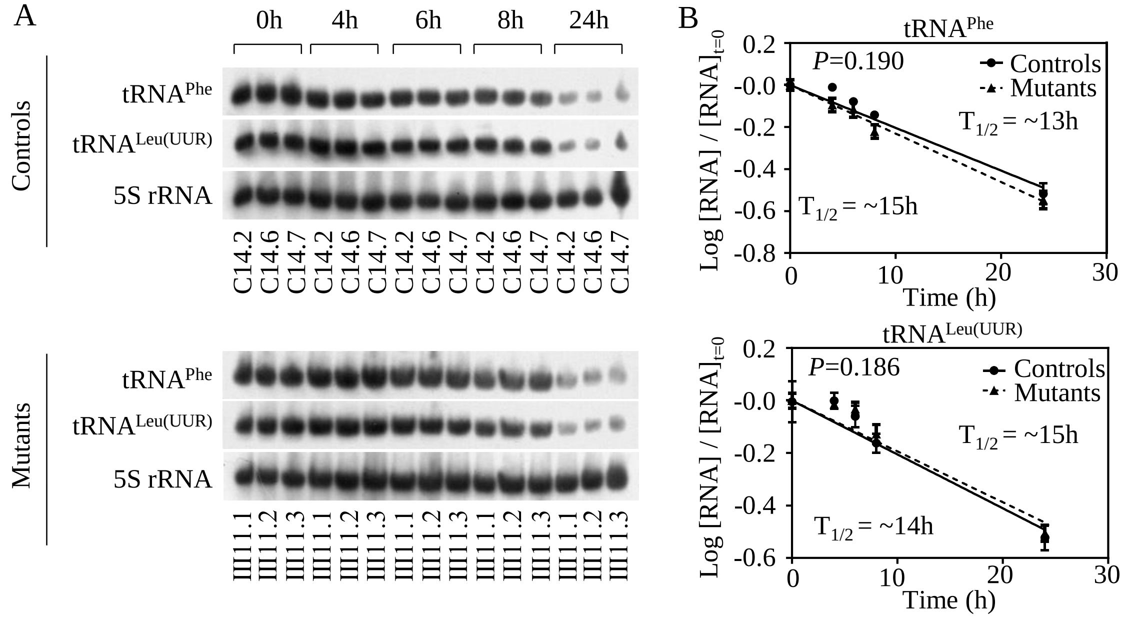

Figure S1. **Decay kinetics of tRNA in three mutant cybrids and three control cybrids.** (A) Mutant and control cell lines were treated with EtBr at various time courses. Total RNAs were isolated, electrophoresed through polyacrylamide gel, electroblotted, and hybridized with DIG-labeled oligonucleotide tRNA probes for $tRNA^{Phe}$, $tRNA^{Leu(UUR)}$ and 5S rRNA, respectively. (B) tRNA levels measured using Image J and expressed as a fraction of the signal obtained from a panel of replicates taken at zero time (the time at which EtBr was added) were converted to logarithms on the assumption of first-order decay kinetics. All hybridization signals were normalized to 5S rRNA as the loading control. The data plotted represent the mean ± 2S.D. (error bars) of three independent experiments. Lines of best fit (least squares method) are shown, R^2 for the two panels (from top left, $tRNA^{Phe}$ to $tRNA^{Leu(UUR)}$) being 0.9257, 0.9397, 0.9257 and 0.9397, respectively.

Supplemental Figure S2

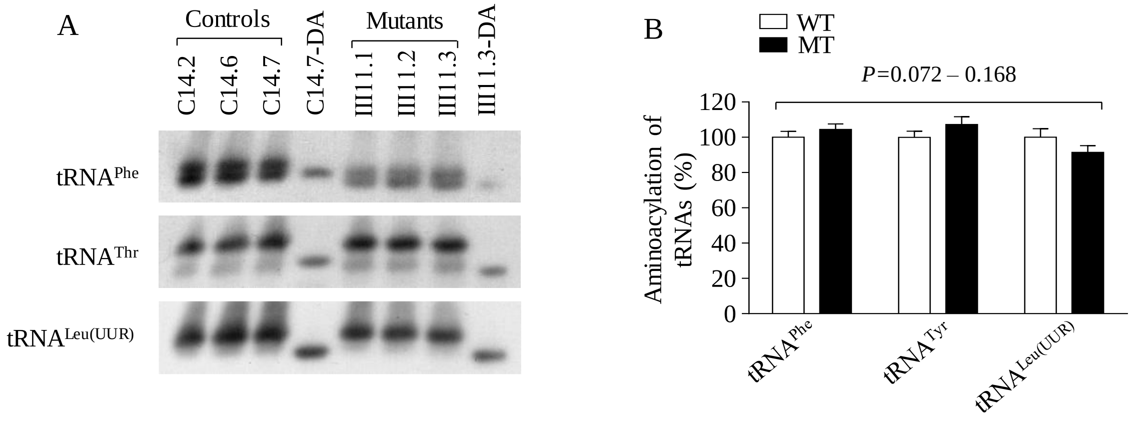

Figure S2. In vivo aminoacylation assays. (A) Twenty micrograms of total cellular RNA purified from six cell lines under acid conditions were electrophoresed at 4˚C through an acid (pH 5.0) 10% polyacrylamide-8 M urea gel, electroblotted, and hybridized with a DIG-labeled oligonucleotide probe specific for the $tRNA^{Phe}$. The blots were then stripped and rehybridized with probes for $tRNA^{Tyr}$ and $tRNA^{Leu(UUR)}$ respectively. The samples from one control (C14.7) and mutant (III11.3) cell lines were deacylated (DA) by heating for 10 min at 60˚C at pH 8.3 and electrophoresed as above. Aminoacylation assays for $tRNA^{Phe}$ were carried out in parallel for aminoacylated and deacylated samples. (B) Qualification of aminoacylated proportions of tRNAs in the mutant and control cell lines. The calculations were based on three independent determinations.

Supplemental Figure S3

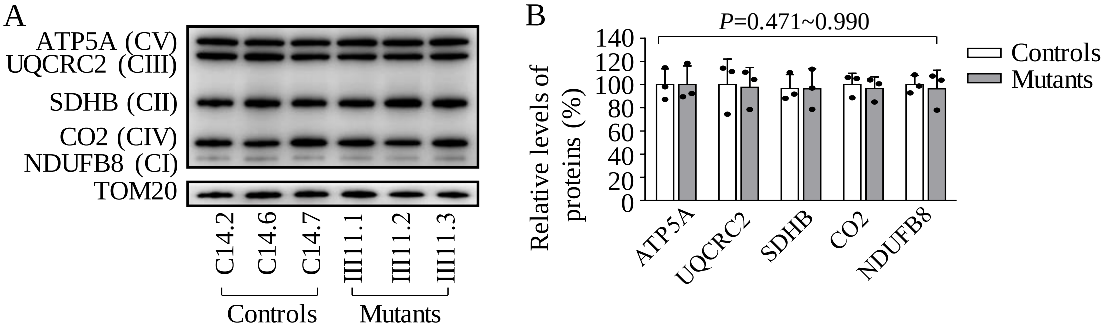

Figure S3 (A) Western blot analysis of OXPHOS subunits (CO2 encoded by mtDNA and other 4 encoded by nuclear genes), with TOM20 as a loading control, respectively. (B**)** quantification of OXPHOS subunits as described above.

Supplemental Figure S4

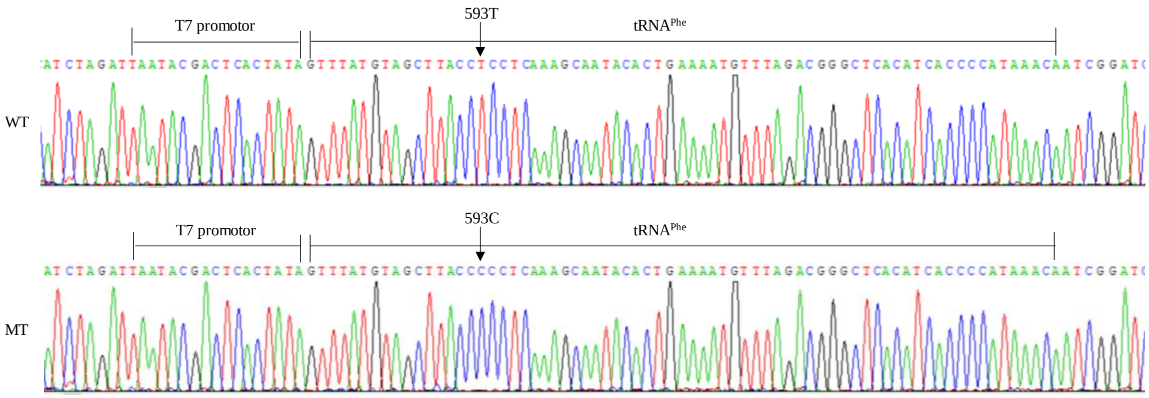

Figure S4. Partial sequence electropherograms of the plasmids used as template for in vitro transcription.

Supplemental Table S1. Usage of phenylalanine codons in human mitochondrial genes and average levels of individual polypeptide in mutant cells related to the average values in the control cells.

Gene	Number of amino acids	Number (Phe)	Proportion (Phe [%])	Relative level of polypeptides (%)
ATP6	227	9	4	NA[a]
ATP8	69	1	1.4	96.34
ND1	318	16	5	34.46
ND2	347	15	4.3	NA
ND3	115	8	7	77.88
ND4	459	20	4.4	64.20
ND4L	99	3	3	NA
ND5	604	38	6.3	34.70
ND6	175	10	5.7	61.27
CO1	514	41	8	27.86
CO2	225	10	4.4	97.65
CO3	261	23	8.8	28.80
CYTB	380	24	6.3	96.34

a. Data not available.

Supplemental Table S2. mtDNA Variants in One hearing-impaired Chinese subject (III-11) and one Chinese control subject (C14)

Gene	position	Replacement	Conservation (H/B/M/X)*	CRS†	III-11	C14	Previously reported‡
**D-Loop**	73	A to G		A	G	G	Yes
	143	G to A		G	A	A	Yes
	146	T to C		T	C	T	Yes
	152	T to C		T	C	C	Yes
	185	G to A		G	A	A	Yes
	207	G to A		G	A	G	Yes
	263	A to G		A	G	G	Yes
	310	T or TC or CTC		T	TC	TC	Yes
	489	T to C		T	C	C	Yes
	514	del C		C	del C	C	Yes
	515	del A		A	del A	A	Yes
	16189	T to C		T	C	C	Yes
	16223	C to T		C	T	T	Yes
	16227	A to G		A	G	G	Yes
	16231	A to G		A	G	A	Yes
	16261	C to T		C	T	T	Yes
	16274	G to A		G	A	A	Yes
	16282	C to T		C	T	T	Yes
	16298	T to C		T	C	C	Yes
	16290	C to T		C	T	T	Yes
	16319	G to A		G	A	A	Yes
	16362	T to C		T	C	C	Yes
	16366	T to C		T	T	T	Yes
**$tRNA^{Phe}$**	593	T to C		T	C	T	Yes
**12S rRNA**	709	G to A	G/G/A/A	G	A	A	Yes
	711	T to C	T/T/A/C	T	C	C	Yes
	750	A to G	A/G/A/A	A	G	G	Yes
	1438	A to G	A/A/A/G	A	G	G	Yes
	1473	C to T	C/C/T/C	C	T	T	Yes
**16S rRNA**	2706	A to G	A/G/A/A	A	G	G	Yes
	3107	DelC		C	Del C	Del C	Yes
**ND2**	4736	T to C		T	C	C	Yes
**ND2**	4769	A to G		A	G	G	Yes
	4833	A to G (Thr to Ala)	T/I/I/L	A	G	G	Yes
	5108	T to C		T	C	C	Yes
	5237	G to A		G	A	A	Yes
	5471	G to A		G	A	A	Yes
**$tRNA^{Ala}$**	5601	C to T		C	T	T	Yes
**CO1**	7028	C to T		C	T	T	Yes
	7600	G to A		G	A	A	Yes
	7621	T to C		T	C	C	Yes
**A6**	8701	A to G (Thr to Ala)	G/G/G/G	A	G	G	Yes
	8856	G to A		G	A	A	Yes

	8860	A to G (Thr to Ala)	T/A/A/T	A	G	G	Yes
	8943	C to T		C	T	T	Yes
**CO3**	9377	A to G		A	G	G	Yes
	9540	T to C		T	C	C	Yes
	9575	G to A		G	A	A	Yes
**ND3**	10398	A to G (Thr to Ala)	T/T/T/A	A	G	G	Yes
	10400	C to T		C	T	T	Yes
**ND4**	10873	T to C		T	C	C	Yes
	11719	G to A		G	A	A	Yes
**ND5**	12705	C to T		C	T	T	Yes
	13395	A to G		A	G	G	Yes
	13563	A to G		A	G	G	Yes
**ND6**	14569	G to A		G	A	A	Yes
**Cytb**	14766	C to T (Thr to Ile)	T/S/T/S	C	T	T	Yes
	14783	T to C		T	C	C	Yes
	14053	G to A		G	A	A	Yes
	15110	G to A		G	A	A	Yes
	15301	G to A		G	A	A	Yes
	15326	A to G (Thr to Ala)	T/M/I/I	A	G	G	Yes
	15746	A to G (Ile to Val)	I/T/I/I	A	G	G	Yes

*Conservation of amino acid for polypepides or nucleotide for RNAs in human (H), bovine (B), mouse (M), and *Xenopus laevis* (X);

†CRS: Cambridge reference sequence;[5]

‡See the online mitochondrial genome database http://www.mitomap.org and http://www.genpat.uu.se/mtDB/
